# Matrix metalloproteinase-9 activity and a downregulated Hedgehog pathway impair blood-brain barrier function in an *in vitro* model of CNS tuberculosis

**DOI:** 10.1038/s41598-017-16250-3

**Published:** 2017-11-22

**Authors:** Sara Brilha, Catherine W. M. Ong, Babette Weksler, Nacho Romero, Pierre-Olivier Couraud, Jon S. Friedland

**Affiliations:** 1 Infectious Diseases and Immunity, Imperial College, London, UK; 2 Centre for Inflammation and Tissue Repair, University College, London, UK; 3 Department of Medicine, Yong Loo Lin School of Medicine, National University of Singapore, Singapore, Singapore; 4 Department of Medicine, Weill Cornell University, New York, USA; 5 Department of Life, Health and Chemical Sciences, Open University, Milton, Keynes, UK; 6 Institut Cochin, Inserm U1016, CNRS UMR8104, Paris Descartes University, Sorbonne Paris Cité, Paris, France

## Abstract

Central nervous system tuberculosis (CNS TB) has a high mortality and morbidity associated with severe inflammation. The blood-brain barrier (BBB) protects the brain from inflammation but the mechanisms causing BBB damage in CNS TB are uncharacterized. We demonstrate that *Mycobacterium tuberculosis* (Mtb) causes breakdown of type IV collagen and decreases tight junction protein (TJP) expression in a co-culture model of the BBB. This increases permeability, surface expression of endothelial adhesion molecules and leukocyte transmigration. TJP breakdown was driven by Mtb-dependent secretion of matrix metalloproteinase (MMP)-9. TJP expression is regulated by Sonic hedgehog (Shh) through transcription factor Gli-1. In our model, the hedgehog pathway was downregulated by Mtb-stimulation, but Shh levels in astrocytes were unchanged. However, Scube2, a glycoprotein regulating astrocyte Shh release was decreased, inhibiting Shh delivery to brain endothelial cells. Activation of the hedgehog pathway by addition of a Smoothened agonist or by addition of exogenous Shh, or neutralizing MMP-9 activity, decreased permeability and increased TJP expression in the Mtb-stimulated BBB co-cultures. In summary, the BBB is disrupted by downregulation of the Shh pathway and breakdown of TJPs, secondary to increased MMP-9 activity which suggests that these pathways are potential novel targets for host directed therapy in CNS TB.

## Introduction

Central nervous system tuberculosis (CNS TB) has high mortality and neurological morbidity even with appropriate treatment^1^. CNS TB is characterized by severe inflammation with destruction of CNS tissues^2^. However, studies on the mechanisms underlying such immunopathology are few.

The blood-brain barrier (BBB) protects the brain, by regulating the transport of substances into the CNS to maintain homeostasis of the microenvironment. It is composed by capillary endothelial cells, surrounded by a basement membrane (comprised of type IV collagen, laminin and fibronectin), pericytes, and the astrocytic perivascular end-feet^3^. The barrier functions of the brain endothelium are dependent on tight junctions (TJ) which comprise of transmembrane tight junction proteins (TJPs), such as occludin, claudin-5 and claudin-3. Cytoplasmic adaptor proteins such as zonula occludens-1 (ZO-1) and ZO-2 connect TJPs to the actin cytoskeleton, allowing TJs to form a seal^4^. This tight seal is broken during CNS inflammation, which is associated with increased BBB permeability and neurological dysfunction^5^.

Astrocyte-derived Sonic hedgehog (Shh) proteins control BBB formation and have crucial roles in maintaining TJ integrity in adult tissues^6,7^. Shh may be associated with the cell plasma membrane or act on target cells over a long range^8,9^. In the target cell, Shh signalling is regulated by Patched (Ptch), a transmembrane protein that inhibits Smoothened (Smo)^10^. This results in translocation of the transcription factor Gli-1 to the nucleus and expression of Gli-1 dependent genes, including TJPs^9^. In the adult CNS, the hedgehog (Hh) pathway is also involved in inhibition of endothelial secretion of chemokines and expression of adhesion proteins required for leukocyte extravasation to the brain^6^.

MMPs are zinc-containing proteases that can degrade components of the extracellular matrix^11^, process cytokines and chemokines^12^, and degrade tight junction proteins^13^. MMP activity is inhibited by non-covalent binding of tissue inhibitors of metalloproteinases (TIMPs) 1–4^14^. In CNS inflammation, increased MMP secretion can affect BBB permeability^15^, with MMP-2 and -9 being associated with BBB breakdown following stroke^16^. In bacterial infections, MMP-8 was shown to be upregulated in a cellular model of meningococcal meningitis^17^, while MMP-9 has been shown to be upregulated in the CSF of children with bacterial meningitis^18^. Increased MMP-9 was also detected in brain biopsies of patients with CNS TB^19^, and elevated MMP-9 concentrations in the CSF were associated with tissue injury and death^20^. Similarly, expression of the collagenase MMP-1 and stromelysin MMP-3 were increased in brain biopsies of patients with CNS TB^21^ and were independent predictors of death^22^. To target MMP dysregulation, host directed therapy with MMP inhibitors has been investigated in CNS inflammatory conditions such as multiple sclerosis^23^. Promising results were also obtained in experimental models of pneumococcal and meningococcal meningitis, where MMP inhibition decreased morbidity and mortality^24,25^.

We hypothesised that Mtb-driven MMP secretion causes disruption of the BBB, contributing to CNS TB immunopathology. We investigated the mechanisms involved in driving BBB disruption in TB using a co-culture model of the BBB. We found that Mtb-stimulation upregulates MMP-9 secretion, which causes type IV collagen and TJP breakdown with associated increase in neutrophil and monocyte transmigration. The Hh pathway was also downregulated, reducing the expression of new TJP. This was due to a decrease in Scube2 activity which affects Shh signalling. Together, our data suggested that the Hh pathway may be a target for host directed therapies reducing inflammation in CNS TB.

## Results

### Stimulation with conditioned medium of Mtb-infected monocytes disrupts a co-culture BBB model and decreases TJP expression

**Figure 1:**
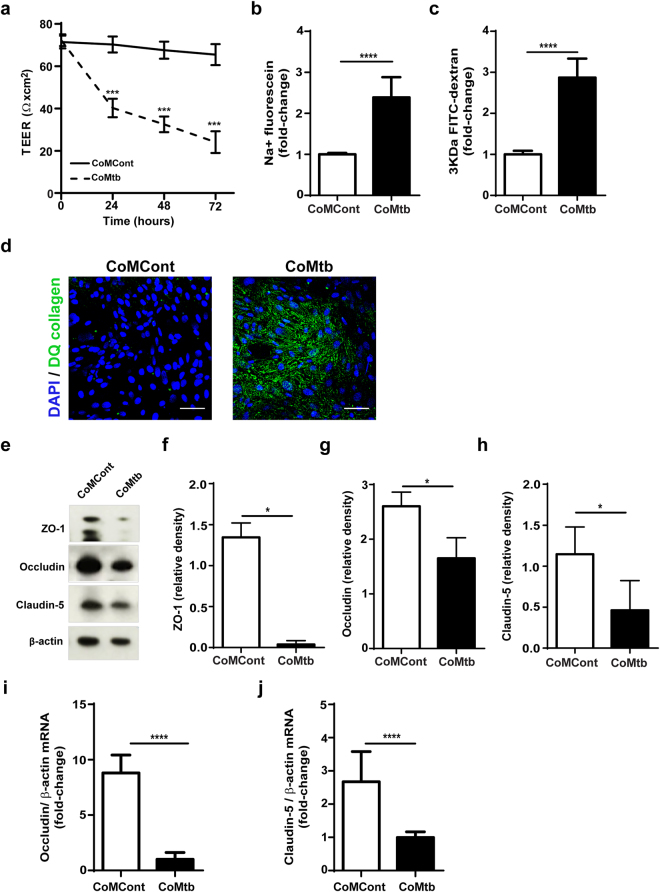
CoMtb causes disruption of the blood-brain barrier. Blood-brain barrier (BBB) co-cultures, consisting of brain microvascular endothelial cells and astrocytes in transwells were stimulated for 72 h with control (CoMCont) or conditioned media from Mtb-infected monocytes (CoMtb). (**a**) Trans-endothelial resistance (TEER; Ω × cm^2^) of BBB cultures with CoMCont or CoMtb stimulation (n = 3). Average background resistance of cell-free coated transwells for each time-point was subtracted. (**b**) Fold-change in BBB permeability to sodium-fluorescein (n = 3). (**c**) Fold-change in BBB permeability to 3KDa FITC-dextran (n = 3). (**d**) Confocal microscopy of the BBB in transwells coated with dye-quenched (DQ) type IV collagen and cells fixed and stained for nucleic acids with DAPI (blue). Green fluorescence depicts areas of collagen degradation. Scale bar: 50 μm. (**e**) Immunoblotting of tight junction proteins, using β-actin as loading control. Membranes were cut according to expected TJP or loading control protein size and each segment incubated with respective antibodies. (**f**) ZO-1, (**g**) Occludin and (**h**) Claudin-5 relative band densities of western blots, normalized to β-actin. Fold-change in (**i**) occludin mRNA (n = 3) and (**j**) claudin-5 mRNA accumulation with CoMtb stimulation (n = 3). β-actin mRNA was used as housekeeping control. Data is represented as mean ± s.d. *p < 0.05 ***p < 0.001 ****p < 0.0001.

We initially tested BBB disruption in CNS TB, by stimulating a human co-culture BBB model with conditioned medium from Mtb-infected monocytes (CoMtb). The model was designed to reflect the *in vivo* findings in man, and consisted of co-cultures of astrocytes and brain microvascular endothelial cells (hCMEC/D3) on type IV collagen coated transwells (Figure S1a). The co-culture BBB model was stimulated with CoMtb or control medium (CoMCont) to mimic *in vivo* cellular networks between astrocytes/endothelial cells and Mtb-infected monocyte-derived cells, which are important in immune responses against Mtb. The BBB model was confirmed to form tight junctions by electron microscopy (Figure S1b), and expressed greater levels of claudin-5 compared with brain microvascular endothelial cells in mono-culture (Figure S1c), which is often used for cellular BBB studies^17,26^. Unstimulated BBB co-cultures had an average trans-endothelial electrical resistance (TEER) of 176 Ω × cm^2^, an average permeability to sodium-fluorescein (≈300 Da; Figure S1e) of 6.5 × 10^−6^ cm/s and an average permeability (Figure S1f) to 3KDa dextran-fluorescein of 8 × 10^−6^ cm/s (Figure S1g). CoMtb stimulation of the co-culture BBB model decreased TEER by 25% at 72 h (p < 0.001; Fig. 1a), while permeability increased significantly to both sodium-fluorescein and 3KDa dextran-fluorescein (p < 0.0001; Fig. 1b,c). In the presence of CoMtb, there was degradation of type IV collagen in the BBB model as demonstrated by increase in fluorescence using dye-quenched (DQ) collagen (Fig. 1D). Protein expression of the TJPs ZO-1, claudin-5 and occludin all decreased after CoMtb stimulation (p < 0.05; Fig. 1e–h). TJP gene expression in microvascular endothelial cells was downregulated during stimulation, with a 7-fold decrease of occludin (p < 0.001; Fig. 1i) and 1.7-fold decrease in claudin-5 mRNA accumulation (p < 0.0001, Fig. 1j).

### *Mycobacterium tuberculosis* drives matrix metalloproteinase secretion in a co-culture BBB model

**Figure 2:**
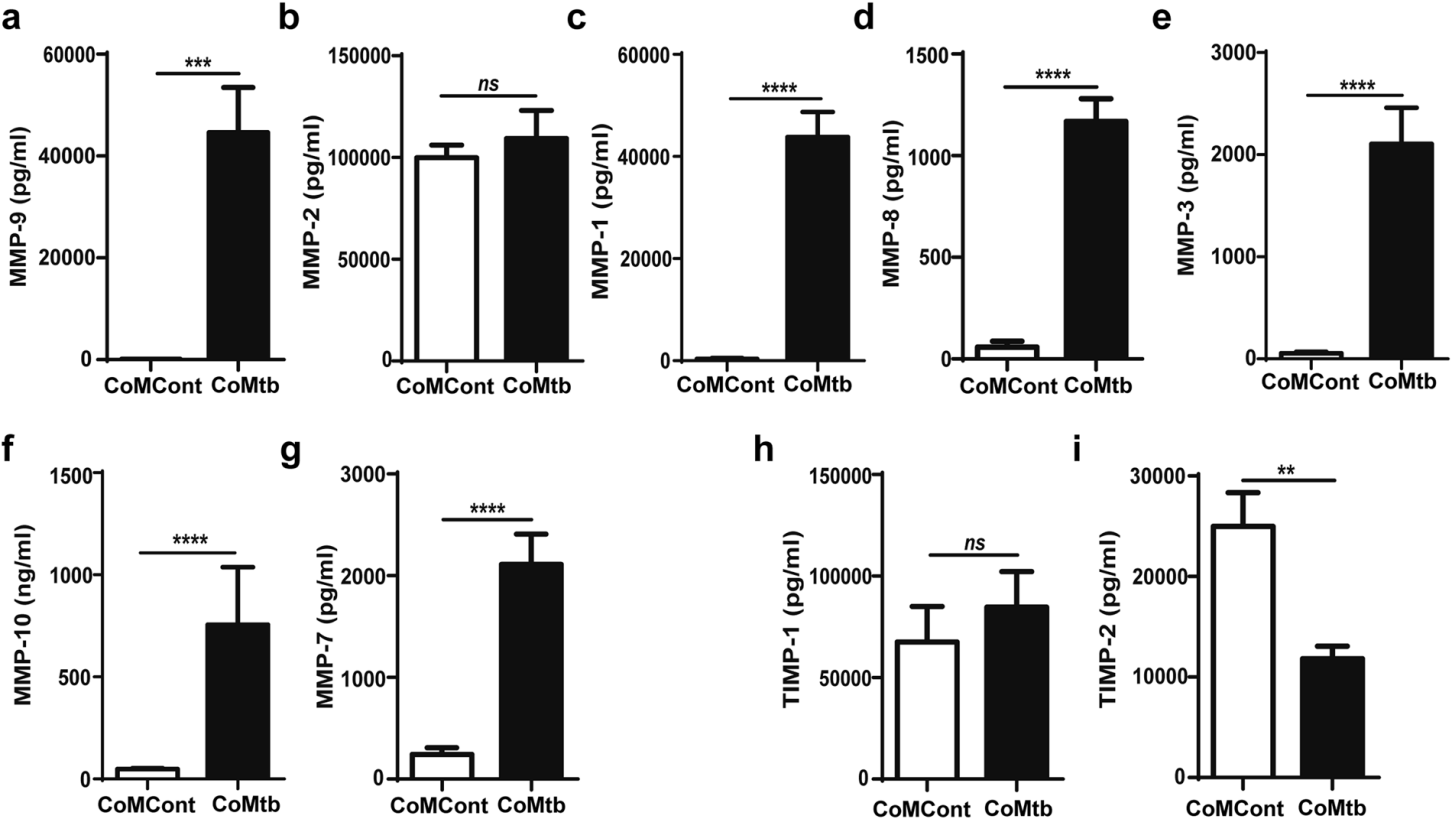
Secretion of MMPs and TIMP-1/2 in the BBB with CoMtb stimulation. Blood-brain barrier (BBB) co-cultures were stimulated for 72 h with control (CoMCont) or conditioned media from Mtb-infected monocytes (CoMtb) and supernatants analysed for (**a**) MMP-9, (**b**) MMP-2, (**c**) MMP-1, (**d**) MMP-8, (**e**) MMP-3, (**f**) MMP-10, (**g**) MMP-7, (**h**) TIMP-1 and (**i**) TIMP-2 (n = 3). Data is represented as mean ± s.d. **p < 0.01; ***p < 0.001 ****p < 0.0001; ns-not significant.

Since increased MMP activity may affect BBB permeability, we next analysed MMP secretion by the co-cultures induced by Mtb-dependent signalling networks. The most upregulated MMP by CoMtb stimulation was the gelatinase MMP-9, the secretion of which increased by 636-fold with CoMtb-stimulation (from 70 ± 14 to 44592 ± 8854 pg/ml; p < 0.001; Fig. 2a), while no significant differences were seen in secretion of the other gelatinase MMP-2 (Fig. 2b). Regarding secretion of collagenases, CoMtb stimulation increased MMP-1 by 124-fold (from 348 ± 194 to 43816 ± 5.1 pg/mL; p > 0.0001; Fig. 2c) and MMP-8 by 19-fold (from 58 ± 29 to 1143 ± 148 pg/ml; p < 0.0001; Fig. 2d). Secretion of the stromelysins which may activate collagenases were increased with MMP-3 secretion upregulated 36-fold (from 56 ± 13 to 2106 ± 353 pg/ml; p < 0.0001; Fig. 2e) and MMP-10 14.7–fold (from 48 ± 5 to 755 ± 282 pg/ml; p < 0.0001; Fig. 2f). Concentrations of the elastase MMP-7 were increased by 8-fold (from 243 ± 67 to 2112 ± 298 pg/ml; p < 0.0001; Fig. 2g). There were no significant differences in secretion of the inhibitor of MMP activity TIMP-1 (Fig. 2h), while secretion of TIMP-2 decreased by 53% in CoMtb-stimulated BBB co-cultures (p < 0.01; Fig. 2i).

**Figure 3:**
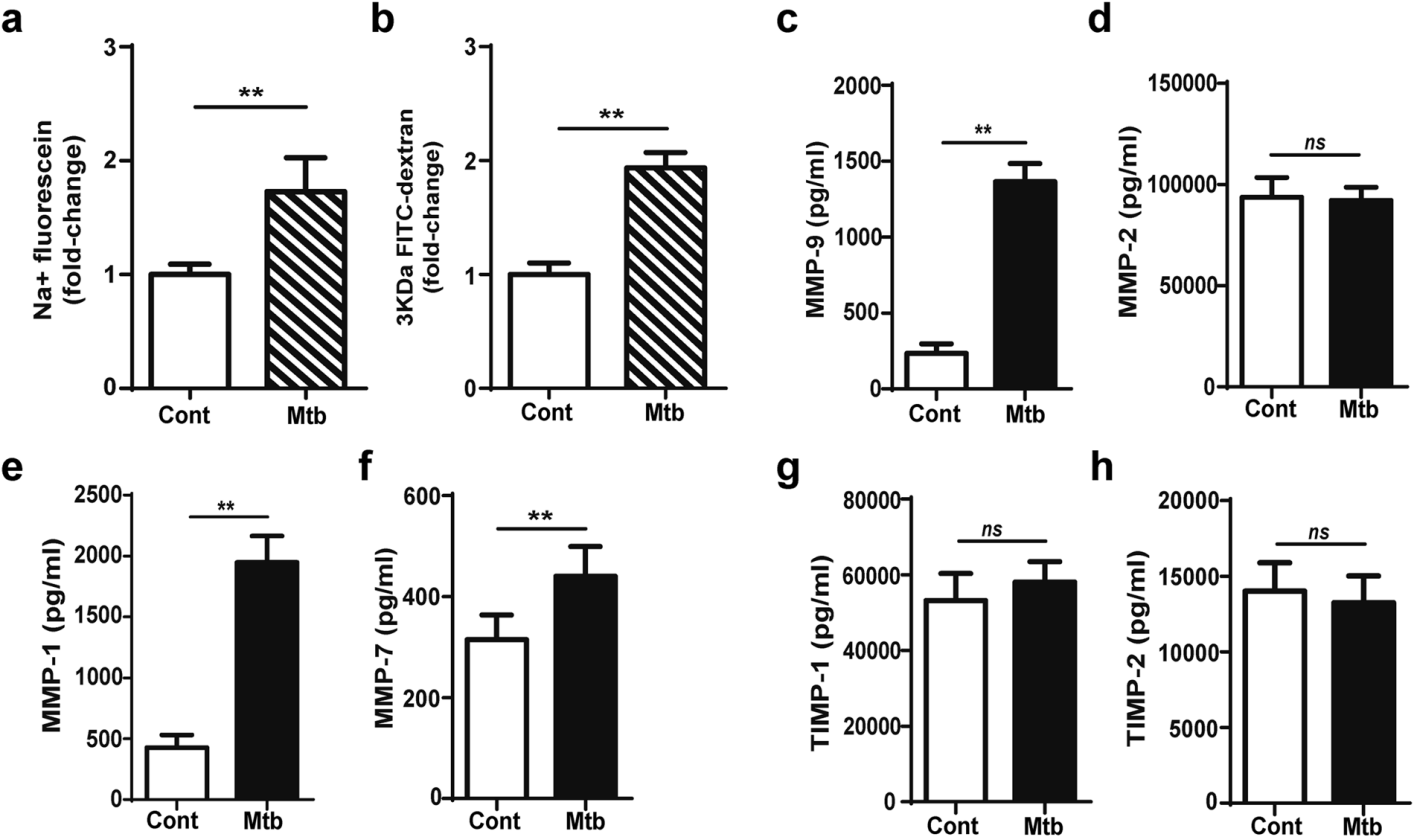
Direct infection by Mtb disrupts the blood-brain barrier. Mtb (MOI 10) was loaded in the apical side of the blood-brain barrier (BBB) and incubated for 72 h. (**a**) Fold-change in BBB permeability to sodium-fluorescein. (**b**) Fold-change in BBB permeability to 3KDa FITC-dextran, relative to unstimulated transwells (n = 3). Concentrations of secreted: (**c**) MMP-9, (**d**) MMP-2, (**e**) MMP-1, (**f**) MMP-7, (**g**) TIMP-1, (**h**) TIMP-2 (n = 3). Data is represented as mean ± s.d. **p < 0.01; ns-not significant.

To further investigate if signalling from monocyte-derived cells was essential or astrocyte activation following Mtb-stimulation was sufficient for BBB disruption, direct Mtb stimulation of the co-culture BBB model was performed. Stimulation with 1.25 × 10^7^ Mtb (MOI = 10) increased permeability to sodium-fluorescein by 83% and to FITC-dextran (3 kDa) by 81% at 72 h (both p < 0.01; Fig. 3a,b). MMP-9 secretion was upregulated by 5-fold (p < 0.01; Fig. 3c) while no differences were detected in MMP-2 (Fig. 3d). MMP-1 secretion was upregulated 3.5-fold (p < 0.01; Fig. 3e), and MMP-7 secretion was upregulated by 40%, (p < 0.01; Fig. 3f). No differences were detected in TIMPs-1/2 (Fig. 3g,h). MMP-3, -8 and -10 concentrations were below the level of detection. MMP upregulation by Mtb was dependent on having a complex model containing astrocytes as the same effects were not observed in cultures of brain microvascular endothelial cells alone (Figure S2). Despite mycobacteria migration occurring across the endothelial layer, and 3.9 × 10^3^ mycobacteria being detected on the basal compartment (Figure S2b), in the absence of astrocytes, Mtb infection did not cause an increase in endothelial barrier permeability (Figure S2c), and no increase in MMP secretion was observed other than for MMP-1 (Figure S2d–f).

### Upregulation of adhesion molecule expression and leukocyte transmigration across the co-culture BBB model with CoMtb stimulation

**Figure 4:**
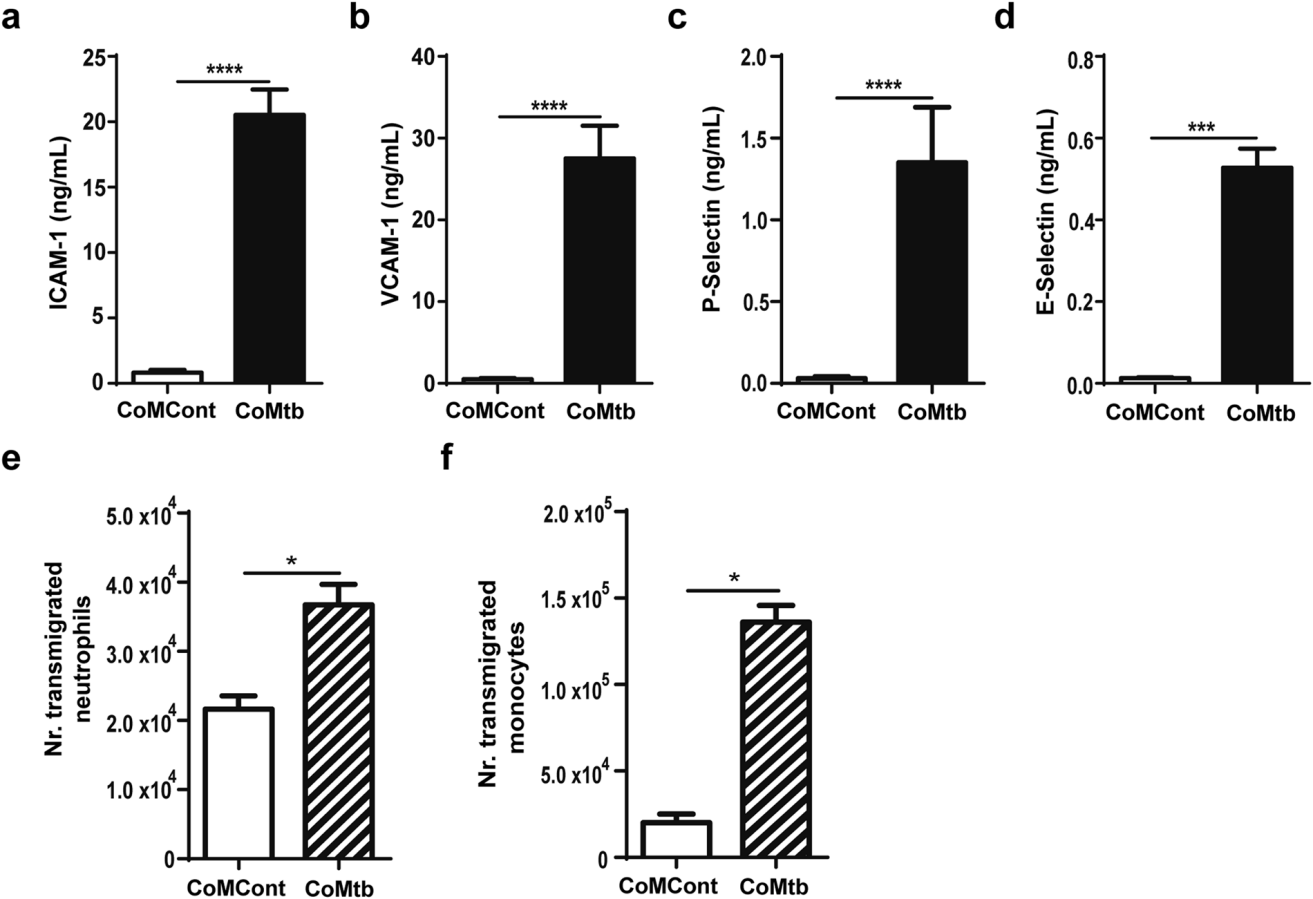
Endothelial adhesion molecules and leukocyte transmigration is increased by CoMtb stimulation. Supernatants from control (CoMCont) or conditioned media from Mtb-infected (CoMtb) stimulated blood-brain barrier (BBB) were collected and analysed for: (**a**) ICAM-1, (**b**) VCAM-1, (**c**) P-Selectin, (**d**) E-Selectin (n = 3). In pre-stimulated or control BBB 5 × 10^6^ monocytes or neutrophils were loaded in the apical side and allowed to transmigrate. Number of transmigrated primary human (**e**) neutrophils and (**f**) monocytes. Figure e and f are representative from 3 independent experiments performed in triplicate. Data is represented as mean ± s.d. *p < 0.05; ***p < 0.001; ****p < 0.0001.

CNS TB is characterised by influx of neutrophils and monocytes across the BBB. These cells are required for host defence but have the potential to cause excess inflammation and tissue damage. For leukocytes to transmigrate across the BBB a series of interactions with endothelial adhesion molecules have to occur. Therefore, we investigated expression of adhesion molecules by the brain microvascular endothelial cells in TB. CoMtb-stimulation led to 24.6-fold increase of endothelial ICAM-1 (from 0.8 ± 0.19 to 20.5 ± 1.8 ng/ml, Fig. 4a), 54-fold increase of VCAM-1 (0.5 ± 0.13 to 27.5 ± 3.8 ng/ml, Fig. 4b), 44-fold increase for P-Selectin (0.03 ± 0.012 to 1.35 ± 0.34 ng/ml, Fig. 4c) and 52-fold increase for E-Selectin (0.01 ± 0.002 to 0.53 ± 0.05ng/ml, Fig. 4d). After pre-stimulation of the co-culture model for 72 h with CoMtb, transmigration of primary human neutrophils increased by 69.5% (Fig. 4e), while monocyte migration increased 5-fold (from 2.49 × 10^4^ monocytes/2 h to 1.24 × 10^5^ monocytes/2 h; Fig. 4f).

### Inhibition of MMP activity prevents BBB co-culture model disruption

**Figure 5:**
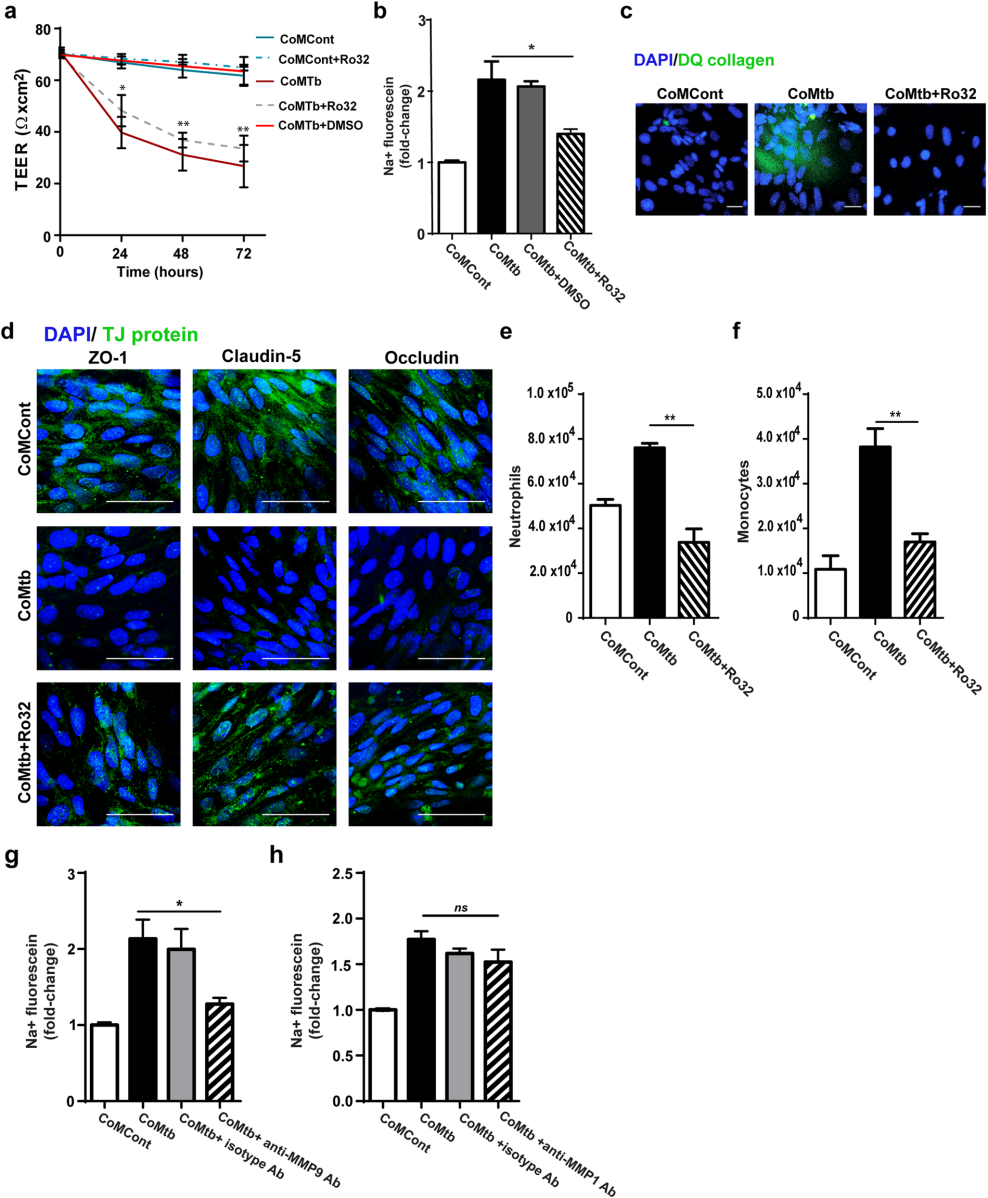
Blockade of MMP-9 activity prevents blood-brain barrier disruption. (**a**) Trans-endothelial resistance (TEER; Ω × cm^2^) of blood-brain barrier (BBB) co-cultures incubated with control (CoMCont), CoMCont +Ro32-3555 (Ro32), conditioned media from Mtb-infected monocytes (CoMtb), CoMtb +Ro32 and CoMtb +DMSO vehicle control (n = 3). Average background resistance of cell-free coated transwells for each time-point was subtracted from measurements. (**b**) Fold-change of flux of sodium-fluorescein relative to control transwells (n = 3). Treatment with 10 μM of MMP inhibitor Ro32 decreased permeability to near control in CoMtb-stimulated BBB. (**c**) Confocal microscopy from transwells coated with dye—quenched (DQ) type IV collagen and stained for nucleic acids with DAPI (blue). BBB were stimulated with CoMCont, CoMtb and/or Ro32-3555 (Ro32). Green fluorescence is released in areas of collagen degradation. (**d**) Confocal microscopy from transwells stained for nucleic acids with DAPI (blue) and for the tight junction proteins ZO-1, claudin-5 and occludin (green). Scale bar: 50 μm. Treatment with Ro32-3555 increased TJP staining. Number of transmigrated (**e**) neutrophils and (**f**) monocytes in CoMtb and CoMtb + Ro32-stimulated BBB. Fold-change in permeability to sodium-fluorescein with addition of: (**g**) 25 μg/ml anti-human MMP-9 neutralising antibodies, or (**h**) 25 μg/ml anti-human MMP-1 neutralising antibodies (n = 3). Figure e and f are representative of 3 independent experiments performed in triplicate. Data is represented as mean ± s.d. *p < 0.05; **p < 0.01.

To investigate whether MMPs were causing disruption of the BBB co-culture, we incubated CoMtb-stimulated BBB with 10 μM Ro32–3555, a broad spectrum MMP inhibitor that blocks activity of collagenases and gelatinases. This abolished CoMtb-dependent BBB co-culture disruption, with TEER remaining similar to CoMCont controls (Fig. 5a), and flux of sodium fluorescein decreasing 37% compared with CoMtb-stimulated barriers (p < 0.05; Fig. 5b), indicating that BBB integrity was maintained with MMP inhibition. Type IV DQ collagen degradation was also decreased with MMP blockade (Fig. 5c). Expression of the TJP ZO-1, claudin-5 and occludin were increased compared to those found following CoMtb stimulation, and abrogated with MMP inhibitor Ro 32–3555 (Fig. 5d).

Blocking MMP activity with Ro32-3555 inhibited CoMtb-stimulated neutrophil and monocyte transmigration across the BBB co-culture by approximately 56% (both p < 0.01) and these migration levels were comparable to baseline (Fig. 5e,f).

Since MMP-9 has been implicated in TJP degradation in neuroinflammation^23,27,28^, we investigated whether MMP-9 was key in driving BBB disruption in CNS TB. Staining for MMP-9 was markedly increased around the brain endothelial cell layer at 72 hours post-stimulation (Figure S3). Pre-treatment with 25 μg/ml anti-human MMP-9 neutralising antibodies decreased permeability by 40% at 72 hours compared to CoMtb-stimulated BBB co-cultures alone (p < 0.05; Fig. 5g). To examine whether BBB disruption was specifically caused by MMP-9, inhibition of MMP-1 was performed, which was the second most upregulated MMP by Mtb-stimulation (over 100-fold; Fig. 2a). No significant changes in permeability were detected for unstimulated and CoMtb-stimulated BBB co-cultures in the presence of anti-MMP-1 neutralising antibodies (Fig. 5h).

### The Hedgehog pathway is downregulated in CNS TB

**Figure 6:**
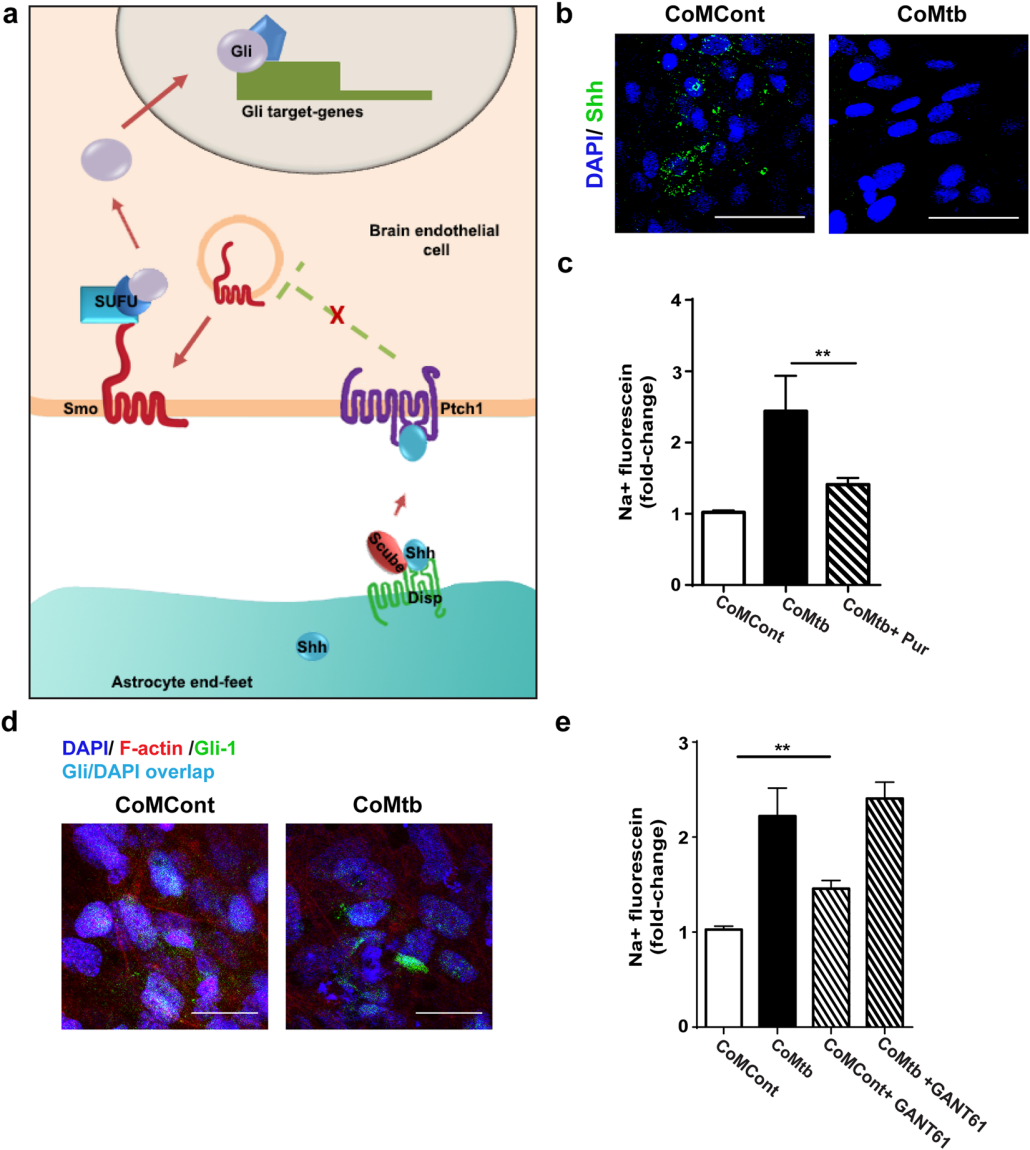
The Shh pathway is downregulated in Mtb-stimulated BBB co-cultures. (**a**) Summarised diagram of the Hedgehog pathway. Shh is produced by astrocytes, where Scube2 is involved in efficient Shh processing and delivery to brain endothelial cells. Shh bound to Ptch1 inhibits its repressor abilities and Smo is translocated to the membrane, leading to inhibition of Suppressor of Fused (SuFu) and activation of Gli transcription factors. Gli accumulates in the nucleus and controls transcription of Hh target genes. (**b–g**) Blood-brain barrier (BBB) co-cultures were stimulated with control (CoMCont) or conditioned media from Mtb-infected monocytes (CoMtb). (**b**) Confocal microscopy of hCMEC/D3 cells stained for nucleic acids with DAPI (blue), and bound Shh (green). Scale bar: 50 μm. (**c**) Fold-change in permeability to sodium-fluorescein of BBB co-cultures stimulated with 1.5 μM smo agonist purmorphamine (n = 3). (**d**) Confocal microscopy of brain endothelial cell layer in transwells stained for nucleic acids with DAPI (blue), Gli-1 transcription factor (green) and actin cytoskeleton (red). Scale bar: 50 μm. (**e**) Fold-change in permeability to sodium-fluorescein for BBB stimulated with 10 μM Gli inhibitor GANT61. Data is represented as mean ± s.d. **p < 0.01.

Since Mtb invasion was also associated with decreased expression of the TJPs claudin-5 and occludin, and the Shh pathway is involved in TJP expression and maintaining TJ integrity^6^ (Fig. 6a), we next evaluated whether Mtb stimulation decreased production of Shh. In CoMtb-stimulated BBB co-cultures, Shh at the surface of brain endothelial cell layer was significantly decreased compared with controls (Fig. 6b). Activation of the Hh pathway by addition of 1.5 μM purmorphamine, which is a Smo receptor agonist, significantly decreased permeability by 42% (Fig. 6c, p < 0.01) indicating maintenance of the BBB integrity.

The transcription factor Gli-1 is an important regulator of TJP gene expression in brain endothelial cells, and is regulated by Shh^29^. Staining of Gli-1 revealed a decreased nuclear location of the transcription factor in CoMtb-stimulated cells (Fig. 6d). Blockade of Gli-1/2 with the specific inhibitor 10 μM GANT-61 caused a 50% increase in permeability to sodium-fluorescein in controls but no additional increase in permeability in CoMtb-stimulated BBB (Fig. 6e). This is likely reflects an inhibition of the Hh pathway by CoMtb stimulation. Higher concentrations of GANT-61 caused significant chemical-associated cell toxicity. Together, these data shows that the Hh signalling pathway is involved in BBB disruption during Mtb infection.

### Mtb-driven MMP-9 downregulates the Hedgehog pathway in CNS TB

**Figure 7:**
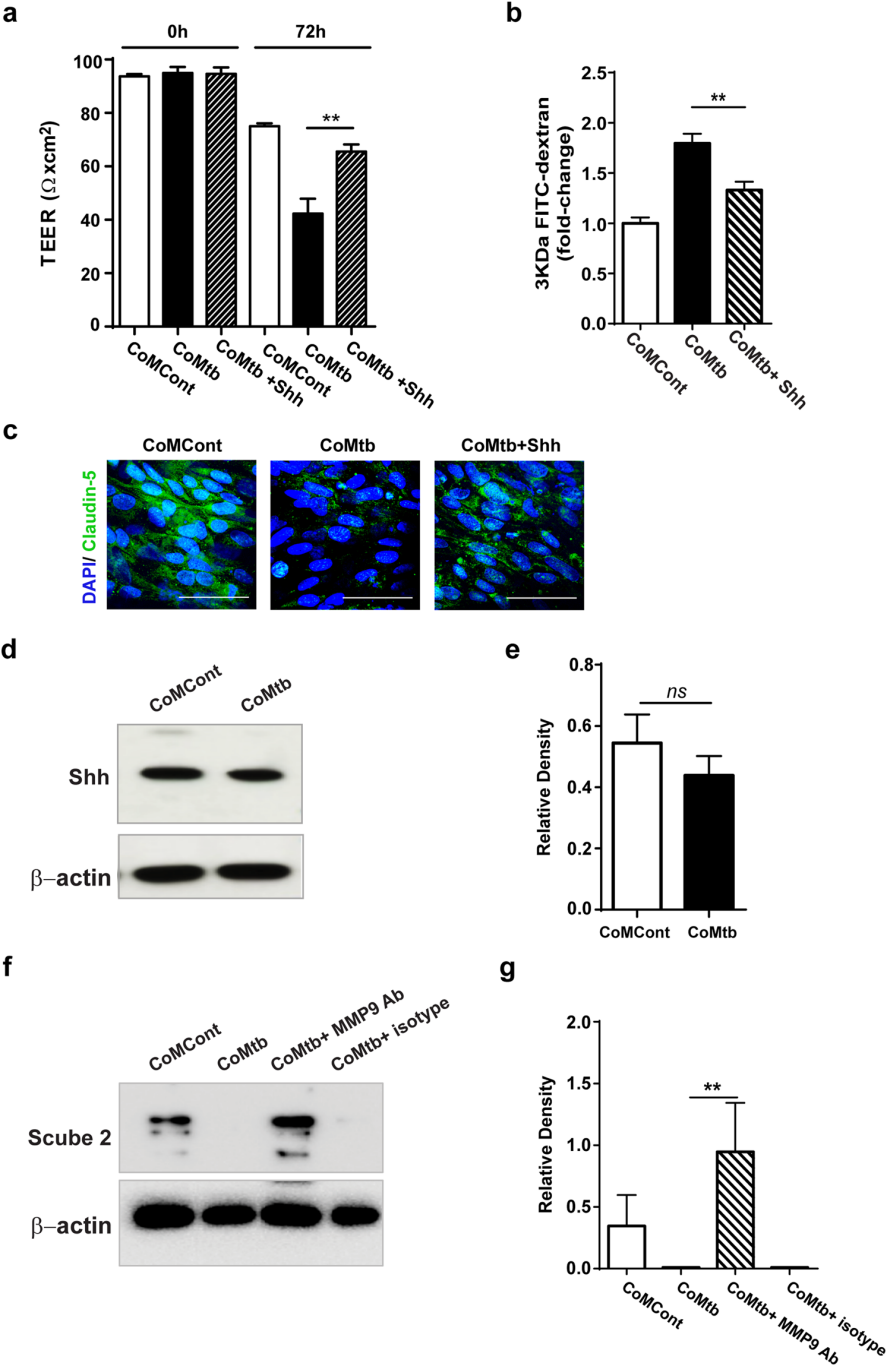
Addition of Shh partially reduced BBB co-culture disruption. Blood-brain barrier (BBB) co-cultures were stimulated for 72 h with CoMCont, CoMtb and treated with 100ng/ml recombinant human sonic hedgehog (Shh). (**a**) Trans-endothelial resistance (TEER; Ω × cm^2^). Average background resistance of cell-free coated transwells was subtracted from measurements. (**b**) Fold-change in permeability to 3KDa FITC-dextran relative to control transwells (n = 3). (**c**) Confocal microscopy of brain endothelial cell layer in transwells stained for nucleic acids with DAPI (blue) and for claudin-5 (green). Scale bar: 50 μm. Cell lysates were used for immunoblots targeting Shh and Scube2. (**d**) Western blot and (**e**) relative band densities of Shh protein levels in CoMCont or CoMtb stimulated human astrocytes (n = 2). (**f**) Representative western blot and (**g**) relative band densities of Scube2 protein levels in astrocytes stimulated with CoMCont or CoMtb and/or MMP-9 neutralising antibody and IgG1 isotype control (n = 2). Membranes were incubated with antibodies against Shh or Scube2 and striped and incubated with loading control, Data is represented as mean ± s.d. **p < 0.01.

To further dissect whether decreased TJP expression during Mtb infection was due to a decreased Hh signalling, we replaced Shh in an attempt to increase TJP expression, and consequently, to increase TEER. The addition of 100ng/ml recombinant Shh improved barrier functions, with a 61% increase in TEER (p < 0.01; Fig. 7a), and a decrease in permeability to 3KDa FITC-dextran (27%; p < 0.01; Fig. 7b). These changes were associated with an increase in TJ proteins ZO-1, claudin-5 and occludin (Fig. 7c; Figure S4). We next analysed Shh production by astrocytes, to investigate if silencing of the Hh pathway in brain microvascular endothelial cells was due to a decreased production of astrocyte-derived Shh. However, no significant difference was found in Shh protein levels in astrocytes stimulated by CoMtb (Fig. 7d,e). These results indicated that it was delivery and not protein expression of Shh that was decreased with Mtb-stimulation.

Scube2 (signal peptide, cubulin domain, epidermal growth factor-like 2) is a secreted glycoprotein that has been implicated in efficient Shh proteolytic processing and secretion^30–32^. Therefore, we analysed Scube2 protein levels in association with astrocytes. In CoMtb-stimulated BBB co-cultures, astrocytes had significantly lower levels of Scube2 than controls (Fig. 7f,g), which will reduce active Shh delivery to endothelial cells during infection. To test if this decrease was dependent on MMP-9, astrocytes were stimulated with CoMtb pre-treated with MMP-9 neutralizing antibodies. MMP-9 neutralization resulted in increased Scube2 protein levels in astrocytes (Fig. 7f,g), indicating that Scube2 expression was dependent on MMP-9.

## Discussion

In this study, we investigated the effects of Mtb infection and monocyte-dependent networks on BBB integrity. We found that Mtb stimulation caused astrocyte activation and upregulation of MMP secretion, which lead to disruption of the BBB co-culture, with breakdown of type IV collagen and the downregulation of the TJPs ZO-1, claudin-5 and occludin. MMP-9 was the only upregulated gelatinase and was found to be the principal MMP causing barrier disruption. MMP-9 concentrations were upregulated 636-fold in response to CoMtb stimulation although just 5-fold after direct Mtb infection demonstrating the importance of networking effects. In addition, CoMtb-stimulation caused at least 19-fold increase of MMP-1, MMP-3 and MMP-8. MMP-9 inhibition by specific blocking antibodies partially abrogated BBB disruption. Our data is consistent with studies in multiple sclerosis, Alzheimer’s disease and bacterial meningitis where MMP-9 drove BBB dysfunction and neuronal injury^22,25,33,34^. VEGF expression has also been implicated in BBB disruption^35^, and a study in paediatric TB meningitis reported that VEGF levels correlated with CSF-serum albumin ratios and mononuclear cell counts^36^. Although we did not analyse VEGF expression in our work, studies have indicated that this growth factor can increase MMP-9 activation^37,38^, which may contribute to the upregulation of MMP-9 we found. The collagenase MMP-1, which we found to be over 100-fold upregulated, did not affect BBB co-culture integrity, as neutralizing MMP-1 did not restore permeability to baseline. MMP-1 is a major driver of tissue damage in pulmonary TB^39^ but the brain ECM contains relatively little fibrillar collagen, therefore in CNS disease, the upregulated MMP-1 may have other non-matrix targets such as IL1-β and proTNF-α^12,40^, which are key pro-inflammatory cytokines in immune responses to Mtb. Increased MMP-9 secretion was relatively unopposed since there was no corresponding increase in its specific inhibitors, TIMP-1 and -2.

An intact non-inflamed BBB restricts leukocyte transmigration into the CNS. We found that in Mtb-stimulated BBB co-cultures, the endothelial adhesion molecules ICAM-1, VCAM-1, P-Selectin and E-Selectin were upregulated over 25-fold and monocyte and neutrophil transmigration across our BBB model were significantly increased compared with controls. Endothelial surface expression of adhesion molecules is required for leukocyte transmigration (arrest, crawling and diapedesis) into the brain parenchyma^41,42^. Leukocyte infiltration is required for an efficient immune response against the invading pathogen however, the unrestricted influx of leukocytes into the CNS, as may occur in CNS TB, has the potential to escalate inflammation causing tissue damage to the surrounding neuronal structures. One limitation of our model was not including pericytes which would pose further technical challenges, such as confocal imaging of triple cultures. Pericytes also have an important role in maintaining blood-brain barrier integrity^43^, and age-associated progressive loss of pericytes was shown to induce BBB leakiness^44^. The role of pericytes in CNS TB pathology is relatively unknown although, a study has reported a loss of pericytes in brain tuberculomas^45^. Therefore, brain microvascular pericytes, or the lack of, may contribute for BBB dysfunction and CNS TB pathology, and should be investigated in future studies.

**Figure 8:**
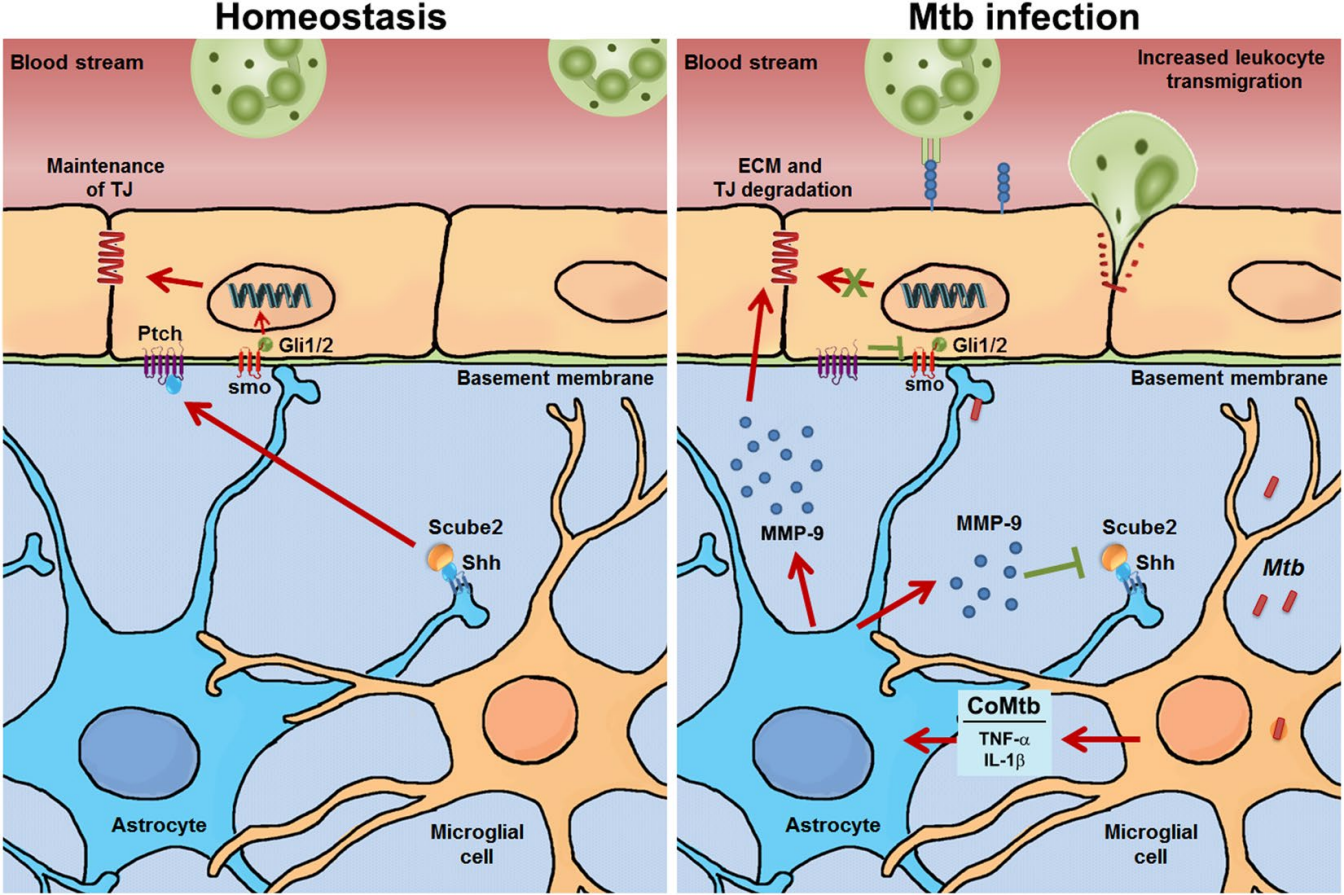
Schematic representation of the proposed mechanism of BBB disruption in CNS TB. In homeostasis (left panel), MMP-9 secretion is low and the human Hedgehog (Hh) pathway is active which maintains blood-brain barrier (BBB) integrity. Astrocyte derived sonic hedgehog (Shh) binds to patched (Ptch), allowing activation of Smoothened (smo), and Gli translocation to the nucleus, leading to the expression of TJP. In *Mycobacterium tuberculosis* infection (right panel), mycobacteria that reach the CNS stimulate microglia and astrocytes, which secrete cytokines such as TNF-α and IL-1β and matrix metalloproteinases. Cellular signalling networks between infected microglial/ monocyte-derived cells (CoMtb), and uninfected astrocytes or microvascular endothelial cells have important roles in driving inflammation. Matrix metalloproteinase (MMP)-9 degrades tight junction proteins (TJPs) and type IV collagen in the basement membrane, increasing BBB permeability. MMP-9 also decreases Scube2 protein levels at the surface of astrocytes, impairing Shh processing and delivery to endothelial cells, downregulating the Hh pathway, and further decreasing TJP expression. Impaired tight junctions together with increased surface expression of endothelial adhesion molecules (e.g. ICAM-1, VCAM-1) result in leukocyte influx into the CNS, causing host immunopathology.

Next, we showed that gene expression of the TJPs claudin-5 and occludin was decreased by Mtb-stimulation and this was associated with a decreased nuclear translocation of Gli-1, demonstrating that the Hh pathway was inhibited during Mtb infection. Consistent with this finding, upregulation of the Hh pathway by addition of the smo agonist purmorphamine or rhShh decreased permeability and increased TEER and TJP expression. The Hh pathway is implicated in BBB integrity in the adult CNS^6,46^ and silencing of the Hh pathway leads to low levels of claudin-3/-5, occludin and ZO-1 and a fragmented basement membrane^6^. Furthermore, upregulation of the Hh pathway, with a smo agonist, was recently shown to reduce BBB disruption in a mouse model of HIV infection^47^. One study using mouse primary cells found that conditioned medium from astrocytes and rhShh increased TJP expression and reported that IL-1β was associated with decreased Shh gene expression by astrocytes^48^. However, we did not detect any significant decrease in Shh protein expression in astrocytes with CoMtb stimulation, which contains IL-1β. Therefore, we hypothesised that an intermediate signalling molecule might be involved in this response. Scube2 has been implicated in active Shh release from the plasma membrane of producing cells, mobilizing it for signalling^31,32,49^. Scube2 may directly extract and transport Shh^30^, while other studies suggested that Scube2 promotes activity of sheddases to release membrane-bound Shh^31,32^. We found that in CoMtb-stimulated BBB co-cultures, astrocytes were associated with significantly lower levels of Scube2, which is required for Shh processing and release from producing cells^32^. This may lead to accumulation of Shh protein by the astrocytes instead of delivery to the target endothelial cells, resulting in the downregulation of the Hh pathway by the BBB. Relatively little is known about the Shh pathway and Scube2. Scube3 is a homolog of Scube2 which has a protein sequence similarity of 90% for the CUB domain and 77% homology for the EGF repeats^50^ and is proteolytically cleaved by MMP-9^51^. Therefore, we propose a model (Fig. 8) in which the marked increased in MMP-9 activity driven by TB might be responsible for Scube2 degradation resulting in decreased Shh delivery to brain endothelial cells and silencing of the Hh pathway. This will ultimately decrease endothelial TJP generation resulting in decreased BBB integrity.

Reducing the inflammatory response in CNS TB has long been suggested in order to decrease the incidence of severe neurological complications and death. Dexamethasone has been tested as an adjunct treatment for CNS TB, and although improving mortality, it failed to improve long-term neurological outcome^52,53^. One study testing the effect on MMP-9 inhibition by dexamethasone, demonstrated that although it was able to significantly inhibit this MMP, it only partially restored ZO-1 expression^54^.

Taken together our data demonstrate that in a model of CNS TB, multiple MMPs are upregulated both in response to TB-dependent networks and in direct infection. We showed that MMP-9 is functionally crucial for causing disruption of the BBB. Besides directly causing breakdown of the basement membrane and TJPs, MMP-9 affects TJP gene expression by suppressing the Hh pathway in brain endothelial cells. MMP-9 may act on Scube2, which affects Shh release from astrocytes and delivery to Ptch at the surface of endothelial cells. Decreased TJP expression prevents repair of the disrupted TJ, favouring immunopathology, therefore stimulation of the Hh pathway may potentially help decrease the inflammation which is responsible for much of the morbidity and mortality in CNS tuberculosis.

## Materials and Methods

### Reagents and antibodies

The following chemical inhibitors were used: Ro32-3555 (Tocris Bioscience, Bristol, UK), purmorphamine and GANT61 (Chemicon, Nottingham, UK). The recombinant human protein Shh (eBiosciences, High Wycombe, UK) was also used. Rabbit anti-human occludin, claudin-5 and ZO-1 antibodies were supplied by Thermo Fisher Scientific (Paisley, UK). Mouse anti-human Shh and MMP-9 antibodies were supplied by Millipore (Hertfordshire, UK). Mouse anti-human β-actin was supplied by Sigma-Aldrich (Dorset, UK). Rabbit anti-human Gli-1, and Scube2 antibodies were supplied by Abcam (Cambridge, UK). Mouse anti-human MMP-1 was supplied by R&D systems. The secondary antibodies alexa fluor 488 conjugated goat anti-mouse IgG (Thermo Fisher Scientific), FITC-conjugated goat anti-mouse IgG (Sigma-Aldrich), DyLight549 conjugated goat anti-mouse Cy5-conjugated goat anti-rabbit (Abcam), HRP conjugated goat anti-rabbit IgG (New England Biolabs, Hitchin, UK) and HRP conjugated goat anti-mouse IgG (Sigma-Aldrich) were also used. A detailed description of the antibodies and dilutions used is shown on Table S1.

### Cell Culture

Primary human astrocytes (HA, ScienCell research laboratories, California, USA) were maintained in T75 flasks pre-coated with poly-L-lysin in complete astrocyte medium (basal medium, astrocyte growth supplement and 2% FBS) with 10 µg/ml ampicillin until the culture was approximately 90% confluent. Cells were used between passages 4 and 10.

The human brain microvascular endothelial cell line hCMEC/D3 (Institute of Cochin, INSERM, Paris, France), which closely mimics primary cells phenotype^55–57^, was maintained in T75 flasks pre-coated with rat tail type I collagen in EBM-2 medium (Lonza, Basel, Switzerland) supplemented with 1.4 μM hydrocortisone, 5 μg/ml ascorbic acid, 1% chemical defined lipid concentrate, 1 ng/ml of basic fibroblast growth factor, 10 mM HEPES, 5% FBS and 10 µg/ml ampicillin, until the culture was 100% confluent. Cells were used between passages 25 and 31. Cells were routinely tested for mycoplasma by PCR.

### BBB cellular model

For co-cultivation, 67,000 astrocytes were seeded in inverted trans-well permeable inserts with 1.1 cm^2^ PET membranes of 5.0 μm pore size (Millicell, Millipore), pre-coated with 150 μg/ml of human type IV collagen (Sigma) in 250 μl of growth medium and let to adhere for 1.5 h at 37 °C and 5% CO_2_. Transwells were inserted in 12-well receiver plates containing 1.2 ml astrocyte growth medium and 50,000 hCMEC/D3 cells were seeded in the apical side in 400 μl endothelial cell growth medium (Figure S1a). The co-culture cells were kept in culture for 6 to 11 days (until TEER levels are above 60 Ohm × cm^2^). Co-cultures were stimulated with conditioned medium from Mtb-infected monocytes (CoMtb, 1:5 dilution) or infected with live, virulent Mtb (MOI 10).

### *Mycobacterium tuberculosis* H37Rv culture

Mtb strain H37Rv was cultured in Middlebrook 7H9 medium supplemented with 10% ADC enrichment medium, 0.2% glycerol, 0.02% Tween 80 with agitation at 100 rpm. Culture growth was monitored with a cell density meter and sub-cultured when the optical density was 1.00, while for infection Mtb cultures were used at an OD 0.6 (mid-log growth phase). For colony forming units (CFU) counting, supernatants and/or cell lysates were seeded in 7H11 agar plates supplemented with 10% OADC enrichment medium, 0.2% glycerol. Plates were incubated at 37 °C for 3–4 weeks.

### Conditioned media preparation (CoMtb)

CoMtb, which contains a pool of cytokines, chemokines and growth factors, secreted by Mtb-infected monocytes^58,59^ was prepared as described^60^. In brief, primary monocytes were purified from single-donor leukocyte cones (National Blood Transfusion Service, London, UK) and seeded at a density of 2.5 × 10^5^ cells/cm^2^ in 60 mm petri dishes and RPMI medium supplemented with 2 mM glutamine and 10 mg/ml ampicillin was added before infecting cells with Mtb strain H37Rv at a multiplicity of infection of 1. Supernatants were collected, filtered through a 0.2 µM polypropylene filter which removes most MMPs^61^ and stored at −20 °C. Control medium (CoMCont) was prepared in a similar manner, in absence of infection.

### Isolation of primary human monocytes and neutrophils from peripheral blood

Peripheral blood from healthy volunteers was used for monocyte and neutrophil isolation. Ethical approval was provided by the Outer West London Research Ethics Committee and work performed in accordance with international standards for research ethics. Written informed consent was obtained from all individuals.

Monocytes were isolated by gradient centrifugation and purified by negative magnetic labelling (MACS monocyte isolation kit II; Miltenyi Biotec Ltd, UK) according to the manufacturer instructions. Neutrophils were isolated by dextran/ saline, followed by gradient centrifugation and erythrocyte hypotonic lysis.

### Transmigration assay

BBB culture and stimulation were performed as described above. Neutrophils or monocytes were gently resuspended in 0.5 μM CellTracker green dye (Thermo Fisher Scientific) working solution and incubated for 20 min on ice. An aliquot of 2 × 10^6^ of primary blood neutrophils or monocytes were left unstained in RPMI with FBS, on ice. Cells were centrifuged and resuspended in DMEM (with 1% FBS, 2% glutamine and 25 mM HEPES) to a final concentration of 5.5 × 10^6^ cells/ml. 150 ng/ml of IL-8 or MCP-1 (chemoattractants for neutrophils and monocytes respectively) in DMEM (with 1% FCS, 2% glutamine and 25 mM HEPES) were added in lower chamber and 200 μl cell suspension (1.1 × 10^6^ cells) in upper chamber. Neutrophils were incubated for 90 min and monocytes for 120 min at 37 °C, 5% CO_2_. Transmigrated cells were gently scraped and collected from the bottom of the trans-well, centrifuged, resuspended in PBS and transferred to FACS tubes. 50 μl CountBright Absolute Counting Beads (Thermo Fisher Scientific) were added to each tube. FACS analysis was performed using 5.5 × 10^6^ unstimulated and unlabelled cells to adjust baseline Forward Scatter, Side Scatter and FL1H settings and gate cells of interest. Number of FL1H positive cells in each condition was assessed by comparing the ratio of bead events to cell events.

### TIMP-1/-2 ELISA

TIMP concentrations were analysed by ELISA (Duoset, R&D Systems) according to manufacturer’s instructions. Lower limits of sensitivity for the Duoset kits are: 21.2 pg/ml for TIMP-1 and 31.2 pg/ml for TIMP-2.

### Luminex Bead Array

Microparticle based multiplex immunoassay were used to detect MMPs and soluble adhesion molecules and were analyzed on the Luminex 200 (BioRad, Hertfordshire, UK). MMPs were analysed using the Fluorokine MAP kit (R&D Systems) according with the manufacturer’s instructions. Lower limits of sensitivity are: 1.1 pg/ml for MMP-1, 12.6 pg/ml for MMP-2, 7.3 pg/ml for MMP-3, 6.6 pg/ml for MMP-7, 16.6 pg/ml for MMP-8, 13.7 pg/ml for MMP-9, and 3.2 pg/ml for MMP-10. All samples were run with appropriate controls and were within the linear range of detection as indicated by the manufacturer. Adhesion molecules were analyzed using the 4-plex human adhesion molecule performance kit (R&D Systems). Lower limits of sensitivity are: 303 pg/ml for ICAM-1, 7.4 pg/ml for E-Selectin, 12.2 pg/ml for P-Selectin and 529 pg/ml for ICAM-1.

### Trans-endothelial electrical resistance (TEER)

TEER measurements were conducted to monitor co-culture barrier properties and barrier integrity, by using STX2 electrode and EVOM2 meter (World Precision Instruments). STX2 electrode was equilibrated in HBSS buffer and the blank measurement was registered. The chopstick STX2 electrode was placed vertically in the wells, allowing the longer (external) electrode to touch the bottom of the dish containing the external culture media while preventing the shorter (internal electrode) from reaching the monolayer at bottom of the tissue culture insert. TEER values were obtained by a 4 point measurement system on trans-well inserts. Type IV coated inserts without cells were used as background controls. TEER was presented as Ohm × cm^2^ (membrane surface area).

### Permeability

BBB permeability to 100 μg/ml of sodium-fluorescein (376.27 Da) or 500 µg/ml of fluorescein-dextran (3 KDa) solutions was assessed. Briefly, 500 μL of fluorescein conjugated molecules were loaded into the apical side of the insert, while the basolateral side contained 2 ml of PBS. Solutions were allowed to permeate the BBB from 30 min to 3 h at 37 °C and 5% CO_2_ and 100 μl samples were collected from the basolateral side for fluorometric analysis using 485 nm excitation and emission filter and 590 nm emission filter. Concentrations were obtained by creating a 6-point standard curve with serial dilutions of the fluorescein conjugated solution. Permeability (Papp) was determined using the equation: Papp (cm/s) = (dQ/dt)/C0*A), where dQ/dt is the rate of permeation (µg/sec), C0 is the initial concentration (µg/ml) and A is surface area of the monolayer (cm^2^).

### Reverse Transcription and Real-time PCR

After 24 hour incubation, hCMEC/D3 cells were lysed with TRI-reagent, total RNA extracted, 1 µg RNA was reverse transcribed and qPCR reactions were performed. Analyses was performed using the Pfaffl comparative Ct method, applying the equation: ratio = E_target_^ΔCt (control^ ^-target)^/ E_reference_^ΔCt (control-target)^; E is the real-time PCR efficiency of one cycle in the exponential phase, calculated according to the equation: E = 10^[−1/slope]^ and the reference gene is β-actin.

### Immunoblotting

Western blotting was used for chemiluminescent detection of ZO-1, occludin, claudin-5, Shh and Scube2. Briefly, SDS denatured proteins were loaded along with a ladder and separated by electrophoresis, using 4–12% Bis-Tris mini-gel and blotted on a nitrocellulose membrane. Membranes was blocked with 0.1% Tween-20, 5% non-fat milk in Tris-buffered saline prior primary antibodies incubation overnight. Membranes were incubated with HRP conjugated secondary antibodies for 1 hour. Finally, membranes were placed in chemiluminescent substrate and exposed to a chemiluminescence film. The membrane was cut according to expected protein size and each segment was incubated with the respective antibodies. When required, membranes were stripped and re-probed with new antibodies. Densitometric analysis of the bands was measured using Scion image analysis and band density of target proteins was normalized to correspondent β-actin bands. Uncropped versions of the western blots are shown as supplementary information.

### Confocal Microscopy

Cells were fixed in 4% paraformaldehyde, blocked with 5% human serum/1%BSA and stained. For intracellular staining, cells were permeabilised with 5% saponin. For cytoskeleton staining, cells were incubated for 30 min with phalloidin-alexa fluor 488. Membranes were carefully removed from inserts and placed between glass slide and cover slip with Fluoroshield mounting medium containing DAPI as the nuclear counterstain. ECM degradation was observed by pre-coating transwells with dye-quenched (DQ) type IV collagen (Life Technologies). Confocal microscopy was performed on a Leica TCS SP5 Confocal equipped with 405 nm diode laser, 488 nm argon laser, 543 nm and 633 nm HeNe lasers and using the Leica Application Suite 2.6.2 software (Milton Keynes, UK). Images were edited using ImageJ software v1.46r (NIH, Maryland, USA).

### Transmission Electron Microscopy

Transwells were rinsed with ice cold PBS and fixed with 0.5% glutaraldehyde in 200 mM sodium cacodylate for 30 min, washed with sodium cacodylate buffer sealed and stored in buffer at 4^o^C until ready to process. Samples were post-fixed in 1% osmium tetroxide and 1.5% potassium ferrocyanide for 1 hour. After washing in water, transwells were incubated in 0.5% magnesium uranyl acetate overnight at 4 ^o^C, dehydrated in ethanol and propylene oxide, and embedded in epon resin, cut into ultrathin sections, and lead citrate was added as a contrast agent. Sections were analysed using an FEI Technai G2 transmission electron microscope, and digital images were captured using Soft Imaging Software.

### Statistical analysis

Data analysis was performed using GraphPad Prism v5.02 (GraphPad software Inc, USA). Data are presented as mean ± standard deviation (s.d.) of 3 independent experiments (n = 3), performed in triplicate unless otherwise stated. Statistical analysis was performed using Mann-Whitney U test or Kruskal-Wallis test with Dunn’s test for pairwise comparisons as appropriate. Differences between variables were considered statistically significant for p-values < 0.05. P-values are represented as follows: *p < 0.05, **p < 0.01, ***p < 0.001 and ****p < 0.0001.

### Data availability

The data that support the findings of this study are available from the corresponding author upon reasonable request.

## Electronic supplementary material

Supplementary information
