# Author Correction: Matrix metalloproteinase-9 activity and a downregulated Hedgehog pathway impair blood-brain barrier function in an *in vitro* model of CNS tuberculosis

**DOI:** 10.1038/s41598-018-31948-8

**Published:** 2018-09-12

**Authors:** Sara Brilha, Catherine W. M. Ong, Babette Weksler, Nacho Romero, Pierre-Olivier Couraud, Jon S. Friedland

**Affiliations:** 1 Infectious Diseases and Immunity, Imperial College, London, UK; 2 Centre for Inflammation and Tissue Repair, University College, London, UK; 3 Department of Medicine, Yong Loo Lin School of Medicine, National University of Singapore, Singapore, Singapore; 4 Department of Medicine, Weill Cornell University, New York, USA; 5 Department of Life, Health and Chemical Sciences, Open University, Milton, Keynes, UK; 6 Institut Cochin, Inserm U1016, CNRS UMR8104, Paris Descartes University, Sorbonne Paris Cité, Paris, France

Correction to: *Scientific Reports* 10.1038/s41598-017-16250-3, published online 22 November 2017

This Article contains an error in Figure 5a, where the key is incorrect. The correct Figure 5 appears below.

**Figure 5:**
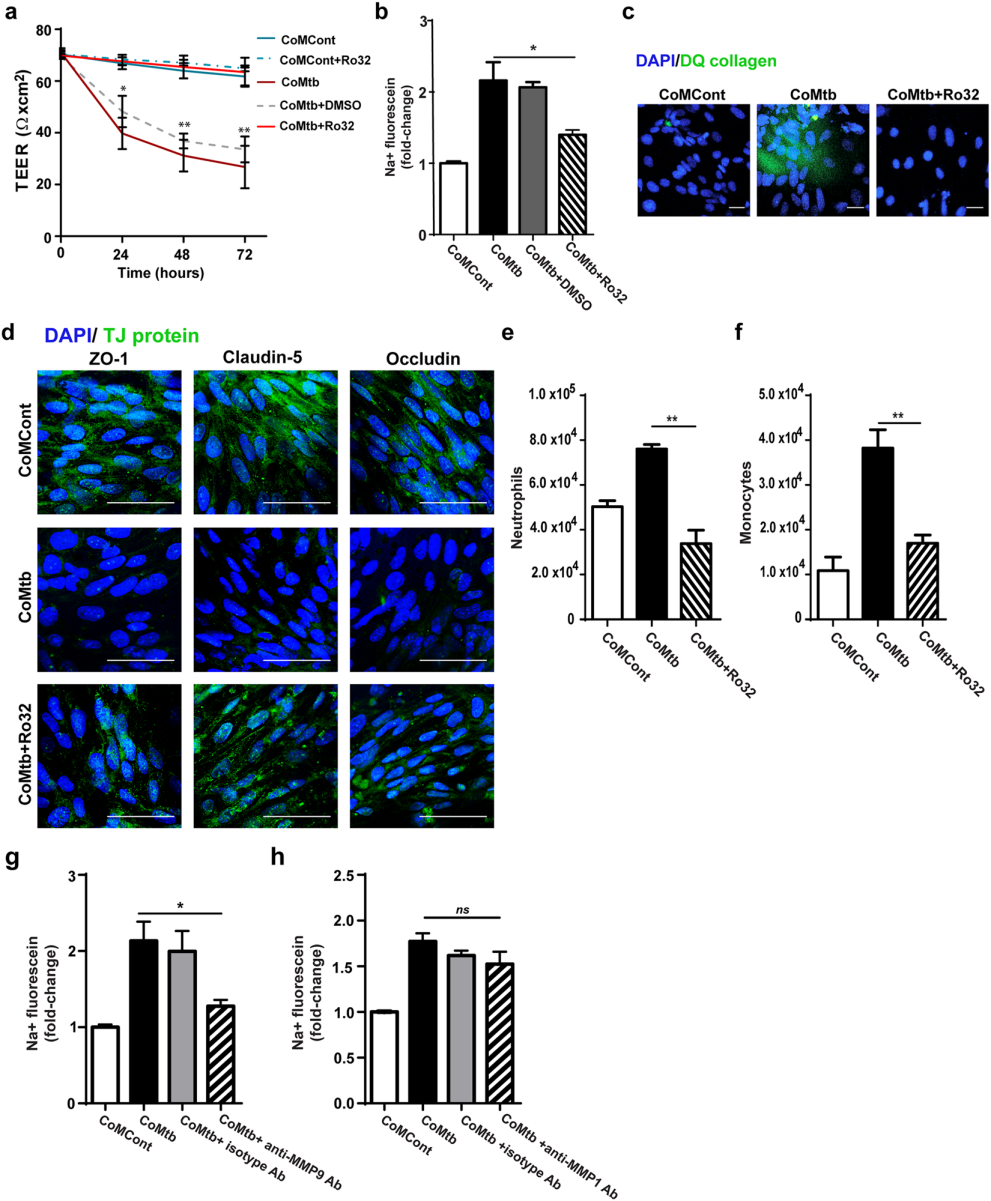
Blockade of MMP-9 activity prevents blood-brain barrier disruption. (**a**) Trans-endothelial resistance (TEER; Ω × cm^2^) of blood-brain barrier (BBB) co-cultures incubated with control (CoMCont), CoMCont +Ro32-3555 (Ro32), conditioned media from Mtb-infected monocytes (CoMtb), CoMtb +Ro32 and CoMtb +DMSO vehicle control (n = 3). Average background resistance of cell-free coated transwells for each timepoint was subtracted from measurements. (**b**) Fold-change of flux of sodium-fluorescein relative to control transwells (n = 3). Treatment with 10 μM of MMP inhibitor Ro32 decreased permeability to near control in CoMtb-stimulated BBB. (**c**) Confocal microscopy from transwells coated with dye—quenched (DQ) type IV collagen and stained for nucleic acids with DAPI (blue). BBB were stimulated with CoMCont, CoMtb and/or Ro32-3555 (Ro32). Green fluorescence is released in areas of collagen degradation. (**d**) Confocal microscopy from transwells stained for nucleic acids with DAPI (blue) and for the tight junction proteins ZO-1, claudin-5 and occludin (green). Scale bar: 50 μm. Treatment with Ro32-3555 increased TJP staining. Number of transmigrated (**e**) neutrophils and (**f**) monocytes in CoMtb and CoMtb + Ro32-stimulated BBB. Fold-change in permeability to sodium-fluorescein with addition of: (**g**) 25 μg/ml anti-human MMP-9 neutralising antibodies, or (**h**) 25 μg/ml anti-human MMP-1 neutralising antibodies (n = 3). Figure e and f are representative of 3 independent experiments performed in triplicate. Data is represented as mean ± s.d. *p < 0.05; **p < 0.01.

