# Supplementary material for: Matrix metalloproteinase-9 activity and a downregulated Hedgehog pathway impair blood-brain barrier function in an in vitro model of CNS tuberculosis
Source: Sci Rep. 2017 Nov 22;7:16031. doi: 10.1038/s41598-017-16250-3 (PMC5700087; doi:10.1038/s41598-017-16250-3)
Supplement: Supplementary file 1 — Supplementary information [file 41598_2017_16250_MOESM1_ESM.pdf]

Matrix metalloproteinase-9 activity and a downregulated Hedgehog pathway impair blood-brain barrier function in an *in vitro* model of CNS tuberculosis

Sara Brilha, Catherine W. M. Ong, Babette Weksler, Nacho Romero, Pierre-Olivier Couraud, Jon S. Friedland

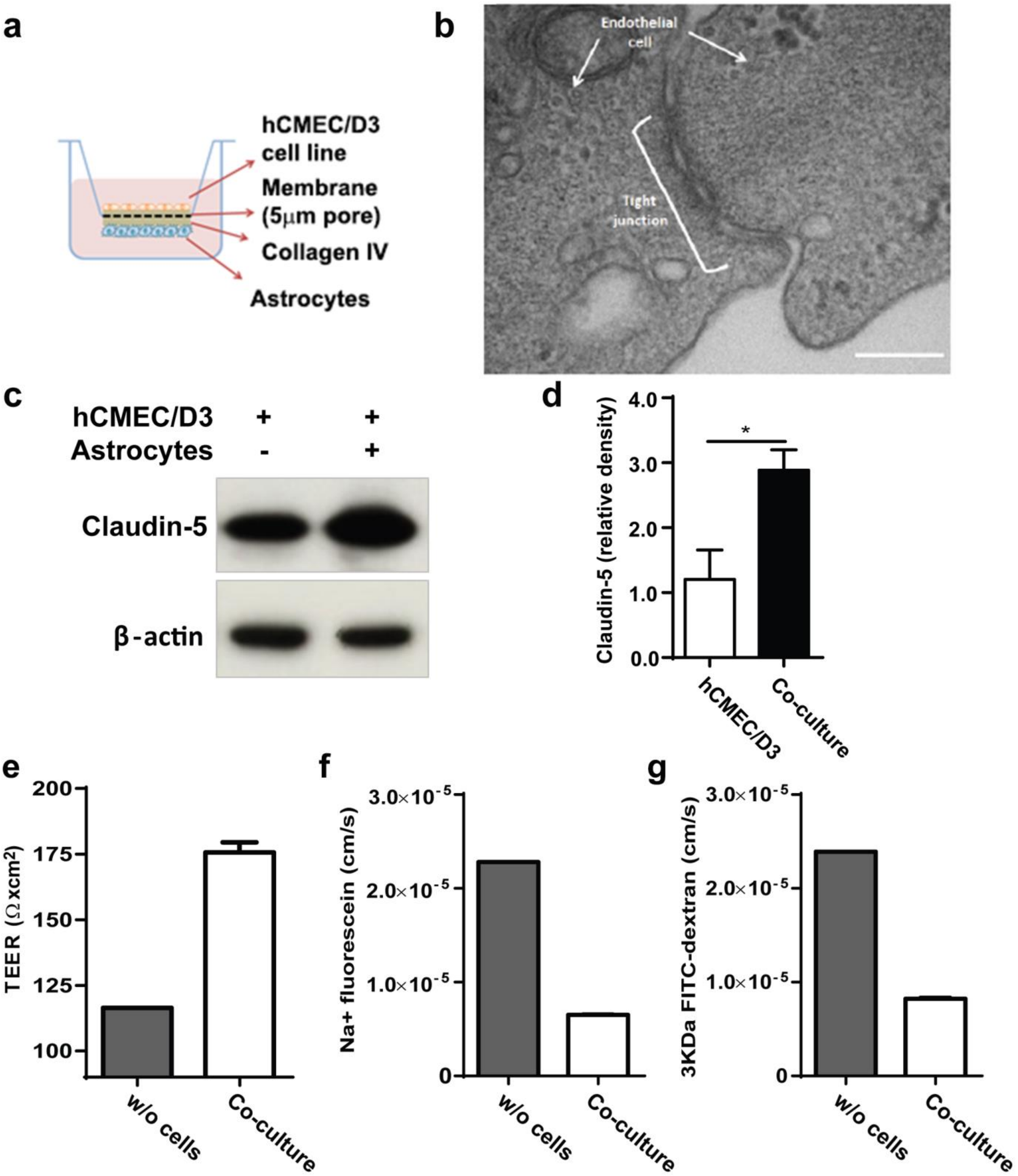

Figure S1- Co-culture BBB model of human hCMEC/D3 cells with primary human astrocytes.

An *in vitro* model was developed by co-culturing the human cell line hCMEC/D3 cells with primary human astrocytes in type IV collagen coated transwells. (**a**) Schematic representation

of the BBB model. (**b**) Establishment of endothelial tight junctions was detected by TEM microscopy. Scale bar: 200nm. (**c**) Western blot and (**d**) relative band density for claudin-5 protein expressed by hCMEC/D3 cells in single culture or in co-culture with primary human astrocytes (n=2). Band densities of claudin-5 were normalized to β-actin. Membranes were cut according with expected claudin-5 and β-actin protein sizes and incubated with respective antibodies. (**e**) Trans-endothelial electrical resistance (TEER) of hCMEC/D3 and astrocyte co-cultures, compared to no cells collagen coated transwells. (**f**) Permeability (Papp) to sodium-fluorescein by hCMEC/D3 and astrocyte co-cultures, compared to no cells collagen coated transwells. (**g**) Permeability (Papp) to 3KDa FITC-dextran by hCMEC/D3 and astrocyte co-cultures, compared to no cells collagen coated transwells. Figures (e-g) correspond to representative experiments. $^*p<0.05$.

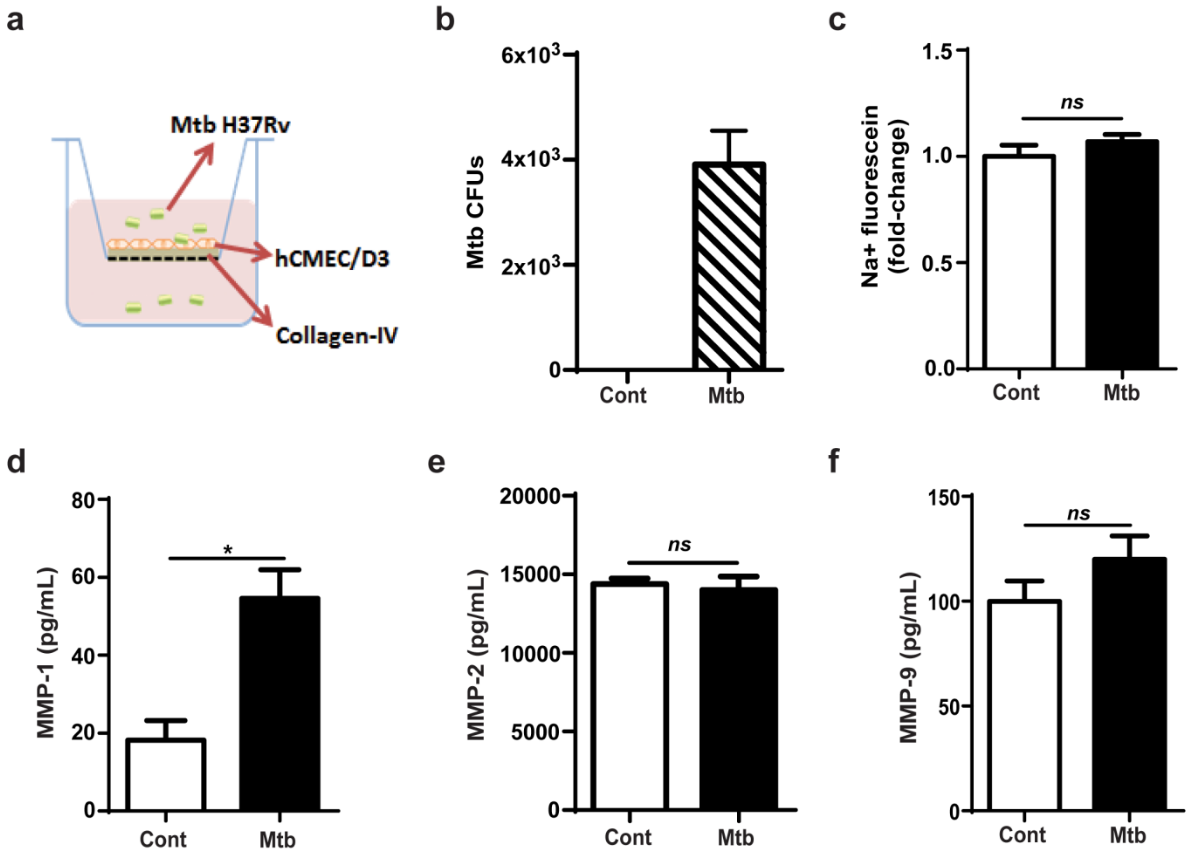

Figure S2- Monoculture of brain endothelial cells do not demonstrate barrier disruption after Mtb-stimulation.

Monoculture of brain microvascular endothelial cells (hCMEC/D3) were performed in type IV collagen coated trans-wells and infected with Mtb (MOI 10) for 72h. (**a**) Schematic diagram of the endothelial cell infection model. (**b**) Colony forming units (CFU) of Mtb collected from the basal compartment. Controls correspond to transwells were an equal volume of PBS was added to apical compartment. (**c**)Fold-change in permeability to sodium-fluorescein (n=3). Secreted concentrations of: (**d**) MMP-1; (**e**) MMP-2; (**f**) MMP-9 were analysed in control and Mtb-stimulated hCMEC/D3 cells (n=3). Data is represented as mean±s.d.. *p<0.05; ns-non significant.

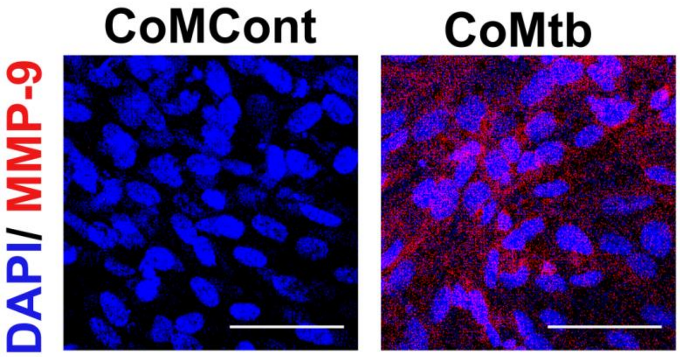

Figure S3- MMP-9 protein expression in endothelial cells is increased in CoMtb-stimulated BBB co-cultures.

Representative confocal microscopy image of brain endothelial cells stained with DAPI for nucleic acids (blue) and mouse anti-MMP-9 Ab with FITC conjugated goat anti-mouse IgG antibodies (red). Scale bar: 50µm.

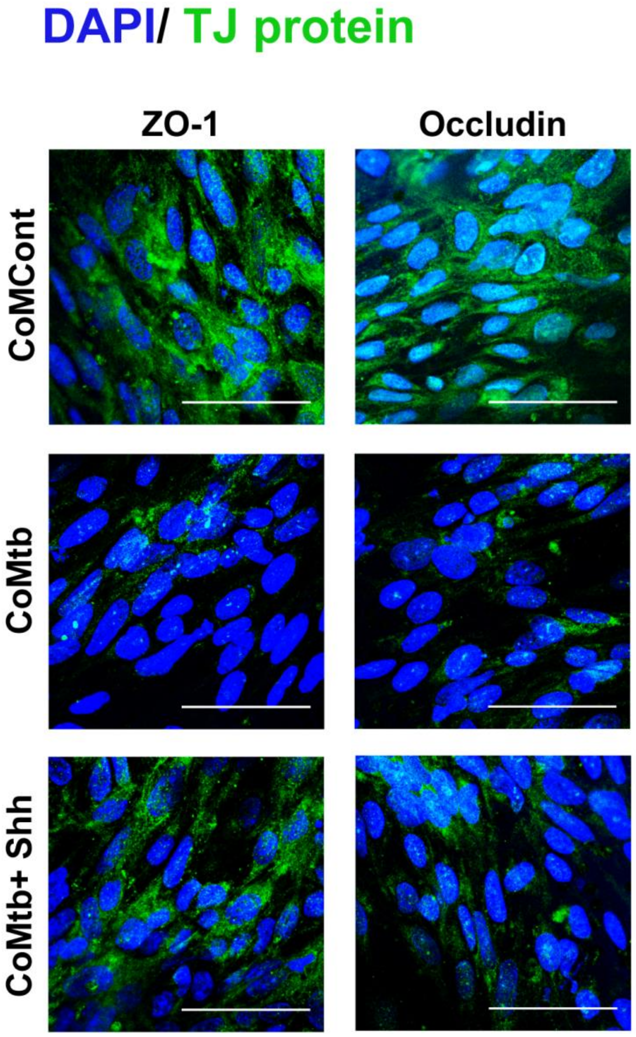

Figure S4- Addition of rhShh increases TJP expression in Mtb-stimulated BBB co-cultures.

Co-cultures were stimulated for 72h with CoMCont, CoMtb and treated with 100ng/ml rhShh. Confocal microscopy from trans-wells stained for nucleic acids with DAPI (blue) and for the tight junction proteins ZO-1, claudin-5and occludin (green). Scale bar: 50µm.

Table S1. Description of antibodies used in the study.

Target	Host	Clone	Concentration / Dilution	Supplier	Catalogue # / RRID
Human ZO-1	Rabbit	Polyclonal	1:500 (WB)<br>1:100 (IF)	Life Technologies	61-7300<br>AB_2533938
Human Occludin	Rabbit	Polyclonal	1:1000 (WB)<br>1:100 (IF)	Life Technologies	711500<br>AB_2533977
human Claudin-5	Rabbit	Polyclonal	1:1000 (WB)<br>1:100 (IF)	Life Technologies	341600<br>AB_2533157
Human Shh	Mouse	5H4	1:1000 (WB)<br>1:100 (IF)	Millipore	MABD175
Human Scube2	Rabbit	Polyclonal	1:500 (WB)	Abcam	ab170373
Human Gli-1	Rabbit	Polyclonal	5 μg/ml (IF)	Abcam	ab49314
β-actin	Mouse	AC-15	1:10000 (WB)	Sigma-Aldrich	A5441
MMP-1	Mouse	36665	25μg/ml (FUNC)	R&D systems	MAB901
MMP-9	Mouse	clone 6-6B	25μg/ml (FUNC)	Millipore	IM09L
Mouse IgG<br>(alexa fluor 488)	Goat	Polyclonal	1:100 (IF)	Life Technologies	A-11001<br>AB_2534069
Mouse IgG<br>(FITC)	Goat	Polyclonal	1:100 (IF)	Sigma-Aldrich	F2772
Mouse IgG<br>(DyLight549)	Goat	Polyclonal	1:100 (IF)	Abcam	ab97008
Anti-rabbit IgG<br>Cy5	Goat	Polyclonal	1:100 (IF)	Abcam	ab6564
Rabbit IgG<br>(HRP)	Goat	Polyclonal	1:1000 (WB)	New England<br>Biolabs	7074
Mouse IgG<br>(HRP)	Goat	Polyclonal	1:5000 (Shh)<br>1:10000 (β-actin)	Sigma-Aldrich	AP127P

RRID: Research Resource Identifier. WB: Western blot. IF: Immunofluorescence. FUNC- Functional assay. HRP: Horse-radish Peroxidase.

1e

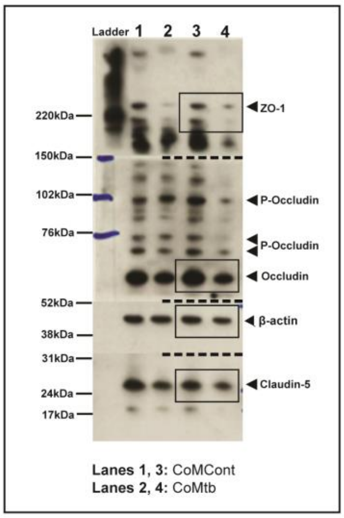

7d

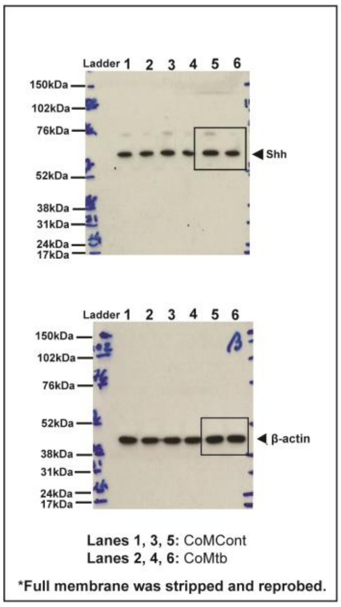

S1

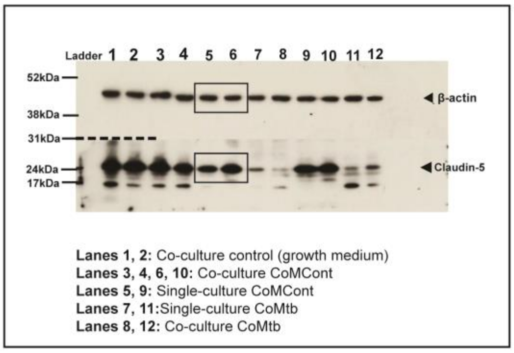

7e

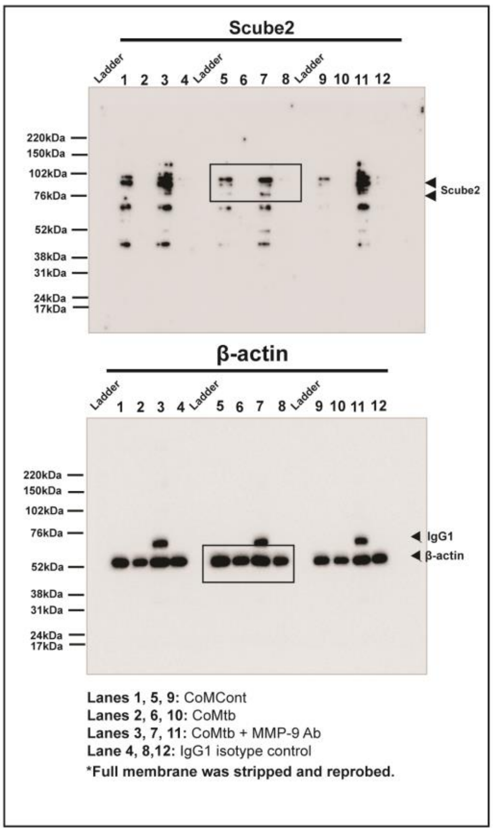

Uncropped versions of Western blots.

Uncropped versions of representative Western blots shown in main figures. Panels are labelled with the corresponding main figure. Dashed lines correspond to areas where membrane was cut to be incubated with different antibodies.
